# Supplementary material for: A network dynamical simulation model for the study of antibiotic resistance in microbial communities
Source: Gut Microbes. 2026 Sep 19;18(1):2734703. doi: 10.1080/19490976.2026.2734703 (PMC13596919; doi:10.1080/19490976.2026.2734703)
Supplement: Supplementary file 1 [file KGMI_A_2734703_SM9053.zip › supplementary_file_1/Figures/ba_delta_gc_frac_n100.pdf]

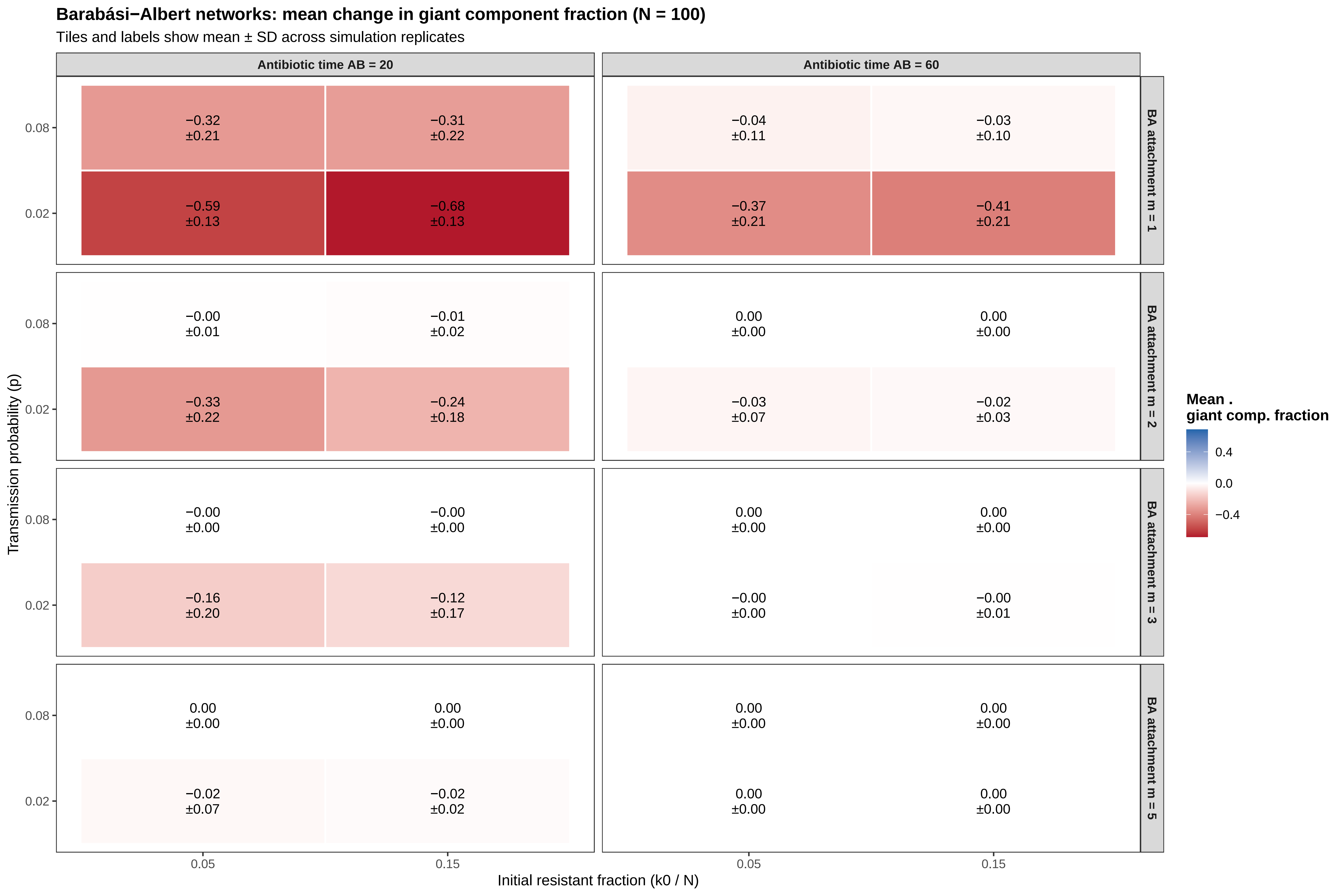
Barabási−Albert networks: mean change in giant component fraction (N = 100)
Tiles and labels show mean ± SD across simulation replicates
Antibiotic time AB = 20
Antibiotic time AB = 60
BA attachment m = 1
BA attachment m = 2
BA attachment m = 3
BA attachment m = 5
−0.32
±0.21
−0.31
±0.22
−0.59
±0.13
−0.68
±0.13
−0.04
±0.11
−0.03
±0.10
−0.37
±0.21
−0.41
±0.21
−0.00
±0.01
−0.01
±0.02
−0.33
±0.22
−0.24
±0.18
0.00
±0.00
−0.03
±0.07
−0.02
±0.03
−0.00
±0.00
−0.16
±0.20
−0.12
±0.17
−0.00
±0.01
−0.02
±0.07
−0.02
±0.02
Transmission probability (p)
0.08
0.02
Initial resistant fraction (k0 / N)
0.05
0.15
Mean .
giant comp. fraction
0.4
0.0
−0.4
